# Trends in Treatment and Survival Among Patients with Glioblastoma in the United States from 2000 to 2020

**DOI:** 10.3390/cancers18172780

**Published:** 2026-08-27

**Authors:** Jianan Chen, Catherine M. Boldig, Qiong Wu, Hannah M. Cardenas, Kaitlyn N. Bernard, Kwadwo Darko, Patrick T. Grogan, Yu Sun, Robert J. Macaulay, Ekokobe Fonkem, Arnold B. Etame

**Affiliations:** 1 Department of Neuro-Oncology, H. Lee Moffitt Cancer Center and Research Institute, Tampa, FL 33612, USA; 2 Department of Neurology, University of South Florida, 2 Tampa General Circle, Tampa, FL 33606, USA; 3 Department of Molecular Medicine, Morsani College of Medicine, University of South Florida, Tampa, FL 33612, USA; 4 Department of Neurosurgery, Geisinger Health System, Danville, PA 17822, USA; 5 Departments of Anatomic Pathology, H. Lee Moffitt Cancer Center and Research Institute, Tampa, FL 33612, USA; 6 Banner MD Anderson Cancer Hospital, University of Arizona, Phoenix, AZ 85006, USA

**Keywords:** glioblastoma, population-based study, treatment patterns, survival, health disparities

## Abstract

Background: Population-level patterns in the real-world use of initial treatment combinations for glioblastoma (GBM) remain poorly characterized. Materials and Methods: We assessed temporal trends in treatment and survival among patients with GBM in the SEER registry. Guideline-concordant multimodal therapy was defined as cancer-directed surgery combined with radiotherapy and chemotherapy. Outcomes were further stratified by extent of resection, including gross total resection (GTR) versus subtotal resection. Results: Among 46,186 patients, 43.5% underwent GTR, 32.0% subtotal resection, and 24.5% no surgery; 71.8% received radiotherapy and 61.3% received chemotherapy. From 2000 to 2020, utilization increased for any surgery (OR/year 1.03, 95% CI 1.028–1.036), radiotherapy (1.01, 1.010–1.017), and chemotherapy (1.08, 1.081–1.088), whereas the likelihood of GTR vs. subtotal resection declined (0.92, 0.915–0.922). Triple therapy increased modestly (1.01, 1.005–1.012) but plateaued at 30%. Older age, non-lobar tumors, unmarried status, and lower income predicted lower odds of triple therapy; after adjustment, calendar year showed a marginal decline in the odds of receiving triple therapy (aOR/year 0.99, 0.99–1.00). Median overall survival improved from 6.0 to 10.0 months, with gains in fixed-time survival. Patients receiving multimodal therapy demonstrated the longest survival overall; within this group, GTR-based multimodal therapy was associated with longer survival than subtotal resection-based multimodal therapy across age strata. Conclusions: Guideline-concordant multimodal therapy was associated with the longest survival. GTR rates did not rise in recent years, and substantial disparities in the deployment of GTR-based multimodal therapy persisted. Efforts to expand equitable access and prioritize maximal safe resection are essential to achieve greater population-level survival gains. These differences should be interpreted cautiously, as SEER cannot distinguish appropriate clinical treatment selection from limited access to care or treatment ineligibility.

## Simple Summary

Glioblastoma is the most common and aggressive type of brain cancer in adults, but little is known about how treatment patterns have changed over time in the general patient population. In this study, we analyzed data from more than 46,000 patients diagnosed with glioblastoma over two decades to understand how treatment use and survival have evolved. We found that while survival has improved modestly, many patients still do not receive the recommended combination of surgery, radiation, and chemotherapy. Older patients, unmarried patients, and those with lower income were less likely to receive this treatment combination. These findings highlight persistent gaps in the delivery of recommended care and the need for strategies to improve equitable access to comprehensive treatment, which may help improve outcomes for patients with glioblastoma at the population level.

## 1. Introduction

Glioblastoma (GBM) is the most common malignant primary brain tumor in adults and is associated with a devastating prognosis, with median overall survival typically less than 1 year and 5-year survival below 10% [1,2]. The pivotal EORTC/NCIC trial established maximal safe resection followed by radiotherapy with concomitant and adjuvant temozolomide as the standard of care, improving median survival from about 12.1 to 14.6 months in selected trial populations [3]. While this protocol improved survival in clinical trials, its translation to real-world populations has yielded only modest gains, with recent data suggesting a plateau in survival outcomes and minimal further improvement from newer modalities [4,5,6].

In routine practice, the delivery of this multimodal standard is highly heterogeneous. Patient-specific factors such as advanced age, poor performance status, and tumor location often limit the feasibility of intensive therapy [7,8]. Furthermore, socioeconomic disparities, health system resources, and regional expertise significantly influence access to specialized care [9,10,11]. Substantial variations exist in the use of surgery, radiotherapy, and chemotherapy across different patient groups and practice settings, with older patients and those from disadvantaged backgrounds being less likely to receive guideline-concordant care [12,13].

Although previous population-based studies have documented the universal adoption of the Stupp regimen and its association with improved survival, they have often focused on single treatment components or selected time periods [14,15]. A comprehensive characterization of how all initial treatment modalities—from no tumor-directed therapy to surgery-based multimodal therapy—are utilized in contemporary, real-world cohorts is lacking. It remains unclear how the adoption of intensive multimodal regimens has evolved over the past two decades, which patient groups are consistently excluded from these advances, and how these patterns relate to population-level survival trends. Few studies have systematically mapped how age, social factors, and detailed treatment combinations interact to shape survival across the entire GBM population.

In this study, we used data from the US Surveillance, Epidemiology, and End Results (SEER) database to examine national trends in initial treatment patterns and survival among patients with glioblastoma diagnosed between 2000 and 2020. We aimed to describe temporal changes in the utilization of surgery, radiotherapy, and chemotherapy; identify factors associated with receipt of multimodal therapy; and assess corresponding trends in overall survival.

## 2. Methods

### 2.1. Study Design and Participants

We conducted a retrospective cohort study using the Surveillance, Epidemiology, and End Results 17 registries database, covering cancer incidence from 2000 through 2020. As the SEER database is de-identified and publicly accessible, this study was exempt from institutional review board approval and patients informed consent.

**Figure 1:**
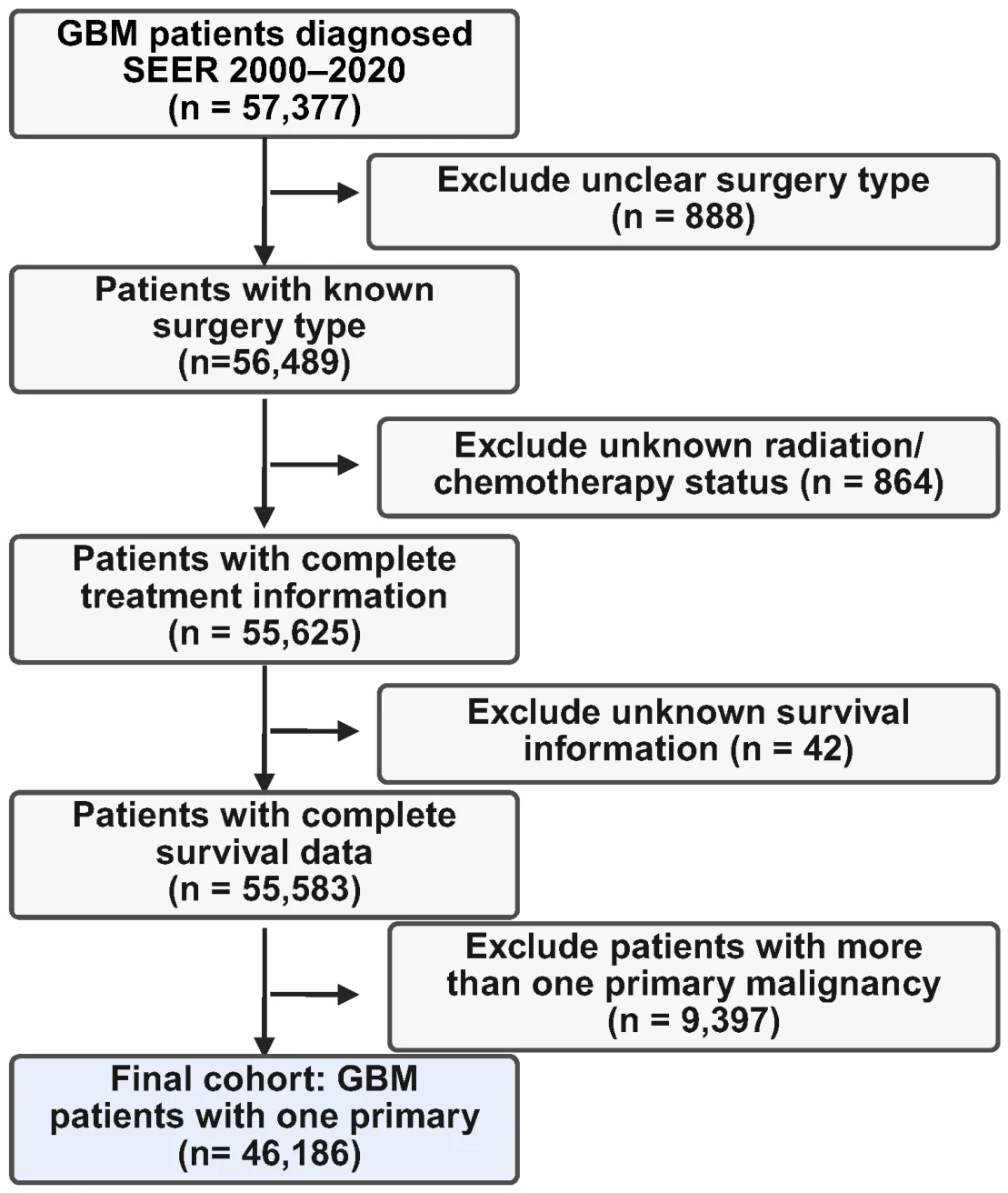
Study profile and cohort selection (SEER 2000–2020). Flow diagram showing the identification of glioblastoma (GBM) cases from the Surveillance, Epidemiology, and End Results (SEER) registry (*n* = 57,377) and sequential exclusions for unclear surgery type, unknown radiotherapy/chemotherapy status, and missing survival information, followed by exclusion of patients with more than one primary malignancy, yielding the final cohort with one primary tumor and complete data (*n* = 46,186).

Adult and pediatric patients with glioblastoma were identified according to SEER-recorded ICD-O-3 histology codes 9440/3, 9441/3, and 9442/3. These diagnoses reflect the histopathologic classification recorded at the time of diagnosis and were not retrospectively reclassified according to contemporary molecular WHO criteria. We included cases where the diagnosed tumor was the first and only primary malignancy. We subsequently excluded records with an unspecified type of surgery, unknown radiotherapy or chemotherapy status, missing survival time, or more than one primary malignancy. The final analytic cohort consisted of 46,186 patients, with the selection process detailed in Figure 1.

### 2.2. Variables and Definitions

Age at diagnosis was categorized into four groups: <18, 18–39, 40–64, and ≥65 years. Patients younger than 18 years were retained to provide a comprehensive population-level description across the full age spectrum but were analyzed as a separate age group given the biological and clinical differences between pediatric and adult GBM. Other demographic variables, including sex, race (White, Black, Other), marital status (Married, Unmarried), and rural/urban residence, were defined according to SEER recodes. Household income, provided by SEER as a county-level attribute, was stratified into three tiers: <$59,999, $59,999–$79,999, and ≥$80,000.

Tumor-specific characteristics included primary site, classified as lobar or non-lobar, and size, grouped as <30 mm, 30–60 mm, or >60 mm. An “Unknown” category was retained for tumor size in descriptive analyses to preserve sample size.

Treatment variables were defined as follows: Surgical resection was categorized as gross total resection (GTR), subtotal resection, or no surgery. “Any surgery” included both GTR and subtotal resection. Receipt of radiotherapy (RT) and chemotherapy (CT) was dichotomized (Yes/No). Gross total resection (GTR) and subtotal resection (STR) were defined according to the SEER “RX Summ-Surg Prim Site” variable, with code 55 representing GTR and code 40 representing partial/subtotal resection. The SEER chemotherapy variable reflects any recorded chemotherapy and does not specify the agent administered. Guideline-concordant multimodal therapy was defined as any cancer-directed surgery combined with radiotherapy and chemotherapy. Analyses further stratified outcomes by extent of resection (gross total vs. subtotal). The calendar year of diagnosis was analyzed both as a continuous variable (for per-year trends) and categorized into four periods: 2000–2005, 2006–2010, 2011–2015, and 2016–2020.

### 2.3. Outcomes

The primary outcome was overall survival (OS), calculated from the date of diagnosis to the date of death from any cause. Living patients were censored at the date of last known follow-up. We reported year-specific median OS with 95% confidence intervals (CIs) derived from Kaplan–Meier estimates. Additionally, fixed-time OS rates at 6, 12, 18, and 24 months were calculated from the Kaplan–Meier curves.

To visualize the interplay of age, treatment, and outcome, we constructed heatmaps. These displayed (i) the proportion of patients within each age group receiving each of the 12 possible treatment combinations (3 surgery types × 2 RT statuses × 2 CT statuses) and (ii) the corresponding Kaplan–Meier median OS for each combination.

### 2.4. Statistical Analysis

Temporal trends in treatment utilization were evaluated using univariable logistic regression, modeling the calendar year as a continuous predictor. We calculated odds ratios (ORs) per year for the receipt of any surgery, RT, CT, and guideline-concordant multimodal therapy. The trend in the propensity for GTR versus subtotal resection was analyzed exclusively among patients who underwent surgery.

Predictors of receiving multimodal therapy were identified using a multivariable logistic regression model, adjusting for age, sex, race, marital status, income, residence, tumor site, tumor size, and calendar year (continuous). Results are presented as adjusted odds ratios (aORs) with 95% CIs. Associations between patient characteristics and survival were assessed with multivariable Cox proportional hazards models, adjusting for age, sex, race, marital status, tumor site, tumor size, surgery extent, RT, CT, income, residence, and diagnosis period. The proportional hazards assumption was verified using Schoenfeld residuals, and no critical violations were found.

All statistical tests were two-sided, with a *p*-value < 0.05 considered significant. Analyses were performed using R software (version 4.5.0).

## 3. Results

### 3.1. Cohort Selection and Baseline Characteristics

From SEER 2000–2020, we identified 57,377 GBM cases; after exclusions (Figure 1), the analytical cohort comprised 46,186 patients. Baseline characteristics are reported for the complete case set (*n* = 46,186; Table 1). Half were aged 40–64 years (50.0%) and 43.0% were ≥65 years; 58.0% were male. Most patients were White (88.5%) and married (62.0%). Tumors were predominantly lobar (71.8%). At diagnosis, 43.5% underwent gross total resection, 32.0% subtotal resection, and 24.5% had no surgery; 71.8% received radiotherapy and 61.3% chemotherapy. Detailed summaries of follow-up duration and censoring status by diagnosis period and age group are provided in Supplementary Table S1. In brief, although censoring was more frequent in the later periods (up to 36.7%), the median follow-up duration in each subgroup surpassed the corresponding median OS, and most patients had already reached the survival endpoint, allowing reliable estimation of median OS across all time intervals.

### 3.2. Age-by-Treatment Heterogeneity

**Figure 2:**
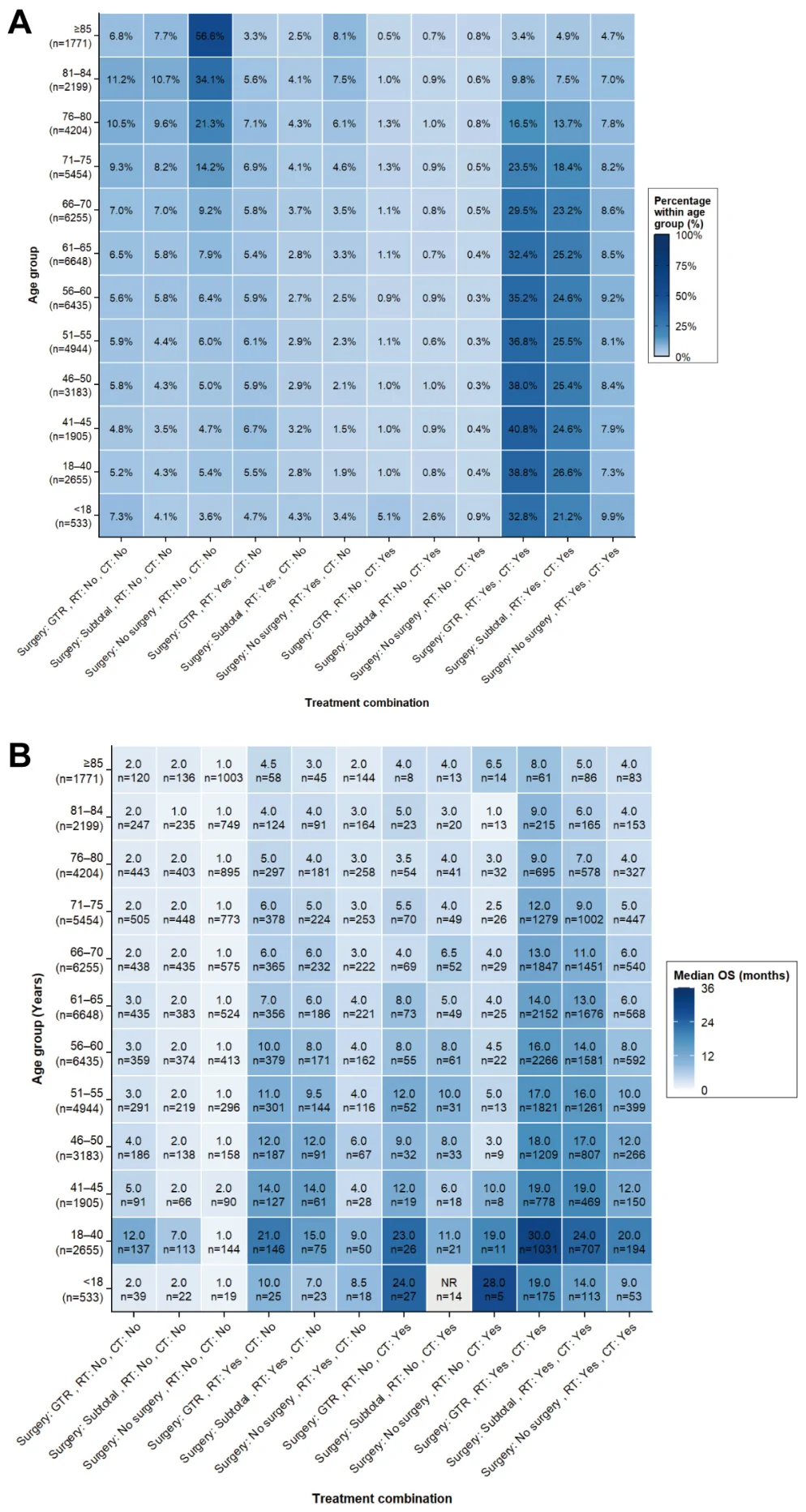
Age-by-treatment patterns and outcomes. (**A**) Heatmap of the distribution of treatment combinations within each age group; values are percentages (%). (**B**) Heatmap of median overall survival (OS, months) for each age-by-treatment cell; each tile displays the median and the sample size (“*n*=”); NR denotes not reached. Treatment combinations are ordered by surgery [Gross total resection (GTR); Subtotal resection; No surgery;] crossed with radiotherapy (RT: No/Yes) and chemotherapy (CT: No/Yes). Age groups are arranged from oldest (top) to youngest (bottom). Color scales indicate percentage (**A**) and survival duration in months (**B**). Abbreviations: GTR, gross total resection; RT, radiotherapy; CT, chemotherapy; OS, overall survival.

Heatmaps of age-specific treatment patterns (Figure 2A) and corresponding median overall survival (Figure 2B) showed a pronounced age–treatment gradient. Within each age group, omission of both radiotherapy and chemotherapy was associated with very poor outcomes (median OS typically 1–5 months), intermediate survival was observed with single- or dual-modality regimens, and the longest survival was consistently observed with triple therapy. Across all adult age strata, triple therapy was associated with the longest survival among available treatment strategies. In patients younger than 18 years, triple therapy remained among the top-performing regimens (median OS 19.0 months), although GTR + RT without chemotherapy showed a median OS of 24.0 months (*n* = 27) and chemotherapy alone showed a median OS of 28.0 months (*n* = 5), both based on small sample sizes. Among adults aged 18–40 years, GTR + RT + CT was associated with the highest survival (median OS 30.0 months), and this age group achieved the longest median survival of any adult age stratum for almost all treatment combinations. The survival associated with intensive therapy attenuated progressively with advancing age: for example, GTR + RT + CT yielded a median OS of 16.0 months in patients aged 56–60 years and 8.0 months in those aged ≥85 years, whereas median OS with no active therapy remained 1–2 months across all age groups.

The use of intensive regimens declined sharply with advancing age. Triple-therapy combinations (with or without gross total resection) accounted for more than two-thirds of initial treatment among patients aged 18–40 years but less than one-fifth among those aged ≥80 years, whereas the proportion receiving no surgery, radiotherapy, or chemotherapy increased from approximately 5–8% in patients younger than 65 years to more than one-third in those aged ≥80 years and over half in those aged ≥85 years.

### 3.3. Temporal Trends in Treatment

**Figure 3:**
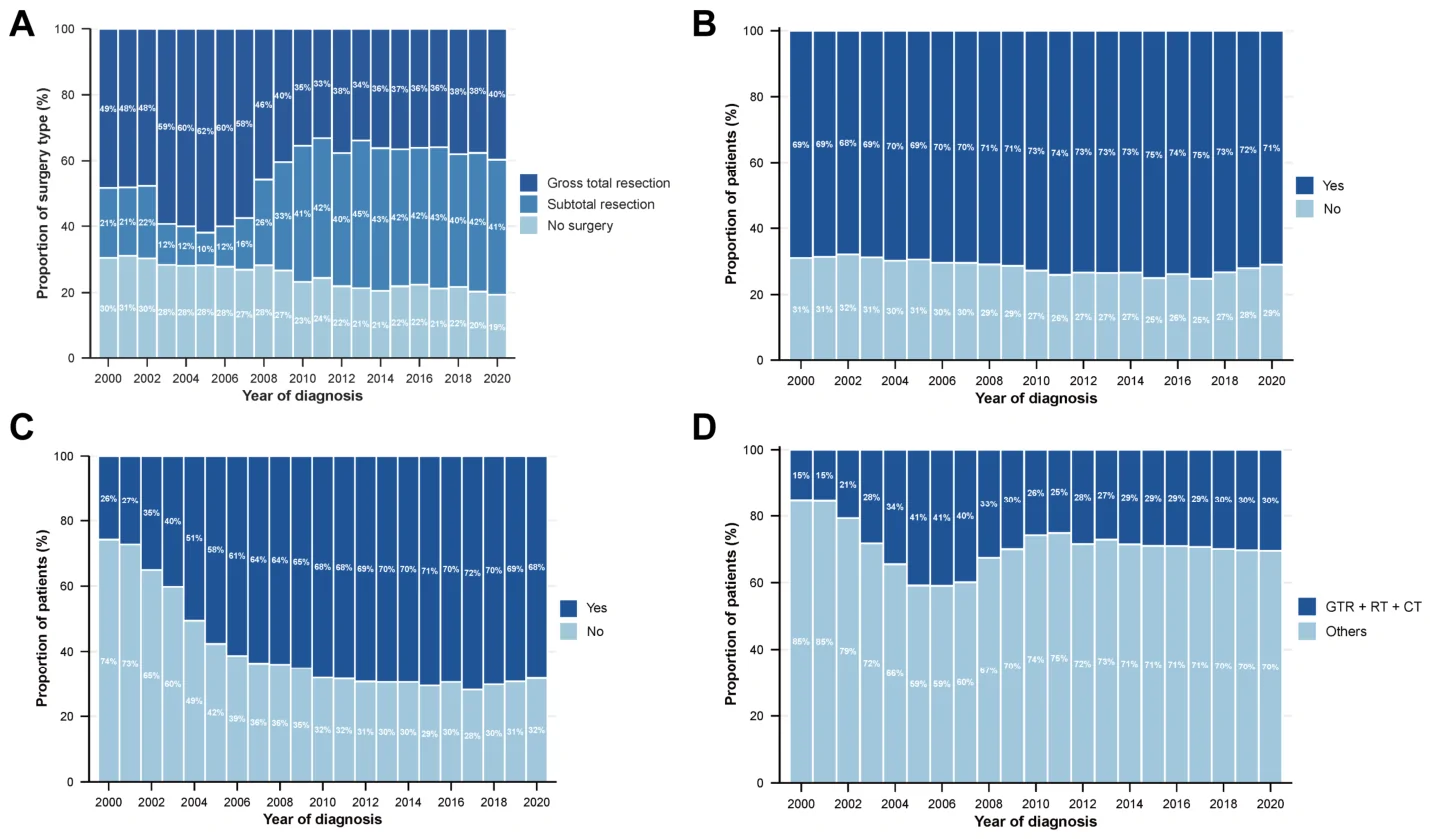
Temporal trends in treatment uptake. Stacked bar charts show the yearly proportion of patients receiving each modality in the analytic cohort: (**A**) surgical management (GTR, Subtotal resection, No surgery,); (**B**) RT (Yes/No); (**C**) CT (Yes/No); and (**D**) GTR-based multimodal therapy (GTR + RT + CT) versus all other combinations.

Between 2000 and 2020, the utilization of the different treatment modalities evolved substantially (Table 2). The odds of receiving any surgery increased by 3% per year (OR 1.03, 95% CI 1.028–1.036). Smaller increases were observed for RT (OR 1.01, 1.010–1.017), while the uptake of CT rose markedly (OR 1.08, 1.081–1.088) (Figure 3B,C). Conversely, the odds of receiving GTR versus subtotal resection decreased over time (OR 0.92, 0.915–0.922) (Figure 3A). The use of GTR-based multimodal therapy (GTR + RT + CT) increased only modestly overall (OR 1.01, 1.005–1.012), plateauing at approximately 30% from the mid-2000s onward (Figure 3D). Most changes in individual treatment utilization occurred during the earlier study years, with radiotherapy, chemotherapy, and surgical patterns becoming relatively stable after approximately 2010. Increasing use of individual modalities did not translate into greater use of the complete GTR + RT + CT combination, particularly given the early decline in GTR relative to subtotal resection.

In the multivariable analysis of factors associated with receiving GTR-based multimodal therapy (Table 3), older age (≥65 vs. <18 years: aOR 0.38, 95% CI 0.30–0.47), non-lobar tumor location (aOR 0.50, 0.47–0.53), and being unmarried (aOR 0.73, 0.69–0.76) were associated with significantly lower odds. Higher household income (≥$80,000 vs. <$59,999: aOR 1.34, 1.22–1.47) was associated with increased odds. After adjustment for these factors, the calendar year was associated with a slight decrease in the likelihood of receiving triple therapy (aOR per year 0.99, 0.99–1.00).

### 3.4. Survival over Time and Determinants of Survival

**Figure 4:**
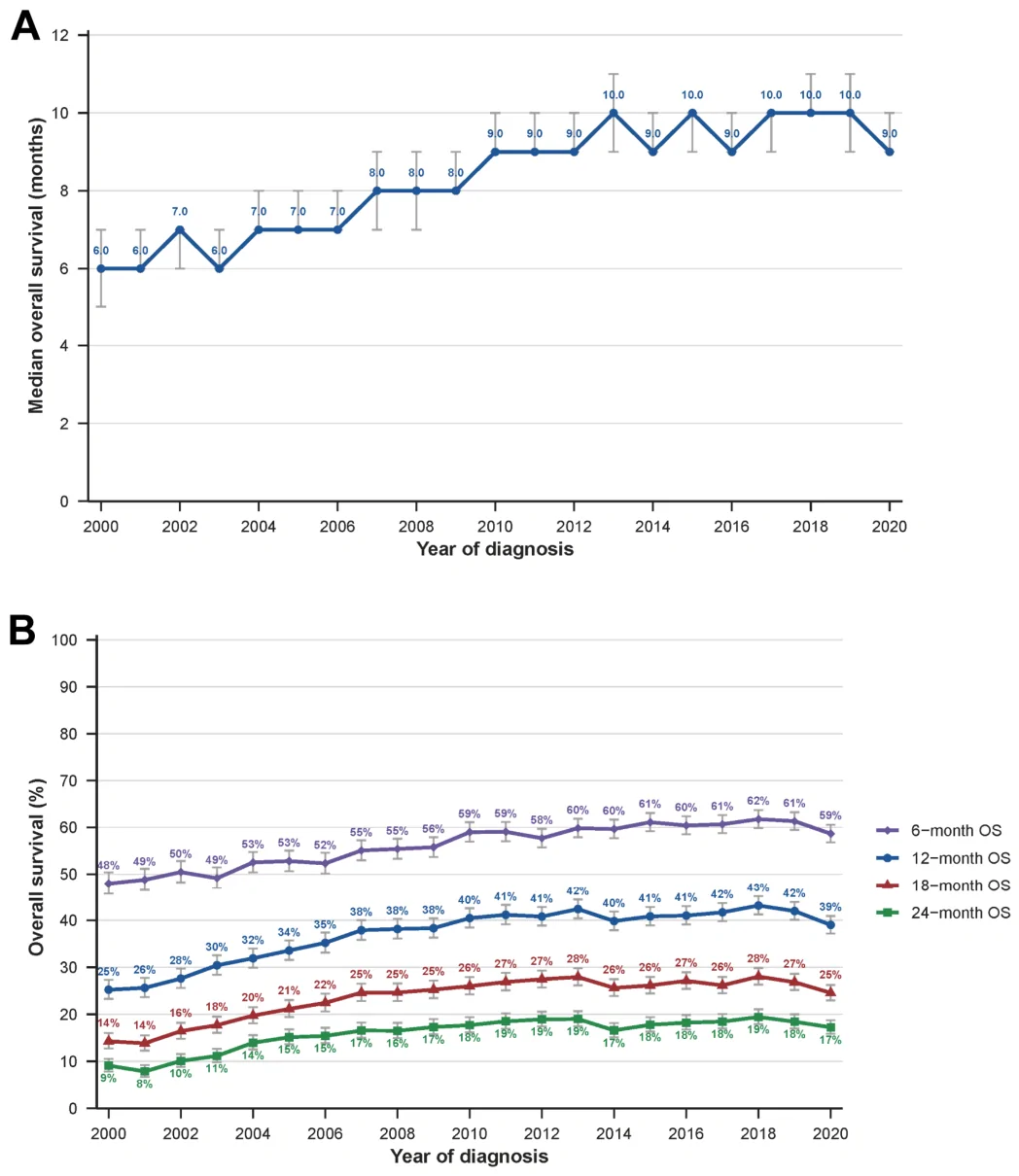
Temporal trends in survival. (**A**) Median OS (months) with 95% confidence intervals (CIs) by year of diagnosis. (**B**) Year-specific overall survival at 6, 12, 18, and 24 months estimated from Kaplan–Meier curves, with error bars showing 95% CIs for each calendar year.

Overall survival improved over the study period. Median OS increased from 6.0 months in 2000 to a peak of 10.0 months between 2013 and 2019 (Figure 4A). Corresponding improvements were seen in fixed-time survival: the 12-month OS rate increased from 25% to 43%, and the 24-month OS rate from 9% to 18% (Figure 4B). Among patients who underwent GTR, median OS increased from 9.0 months (95% CI, 9.0–10.0) in 2000–2005 to 12.0 months (95% CI, 11.0–12.0) in 2006–2010 and 14.0 months (95% CI, 14.0–15.0) in 2011–2015, remaining at 14.0 months (95% CI, 14.0–15.0) in 2016–2020 (Supplementary Table S2). These findings indicate that the improvement in survival following GTR occurred predominantly during the earlier study periods, followed by an apparent plateau in the most recent decade.

**Figure 5:**
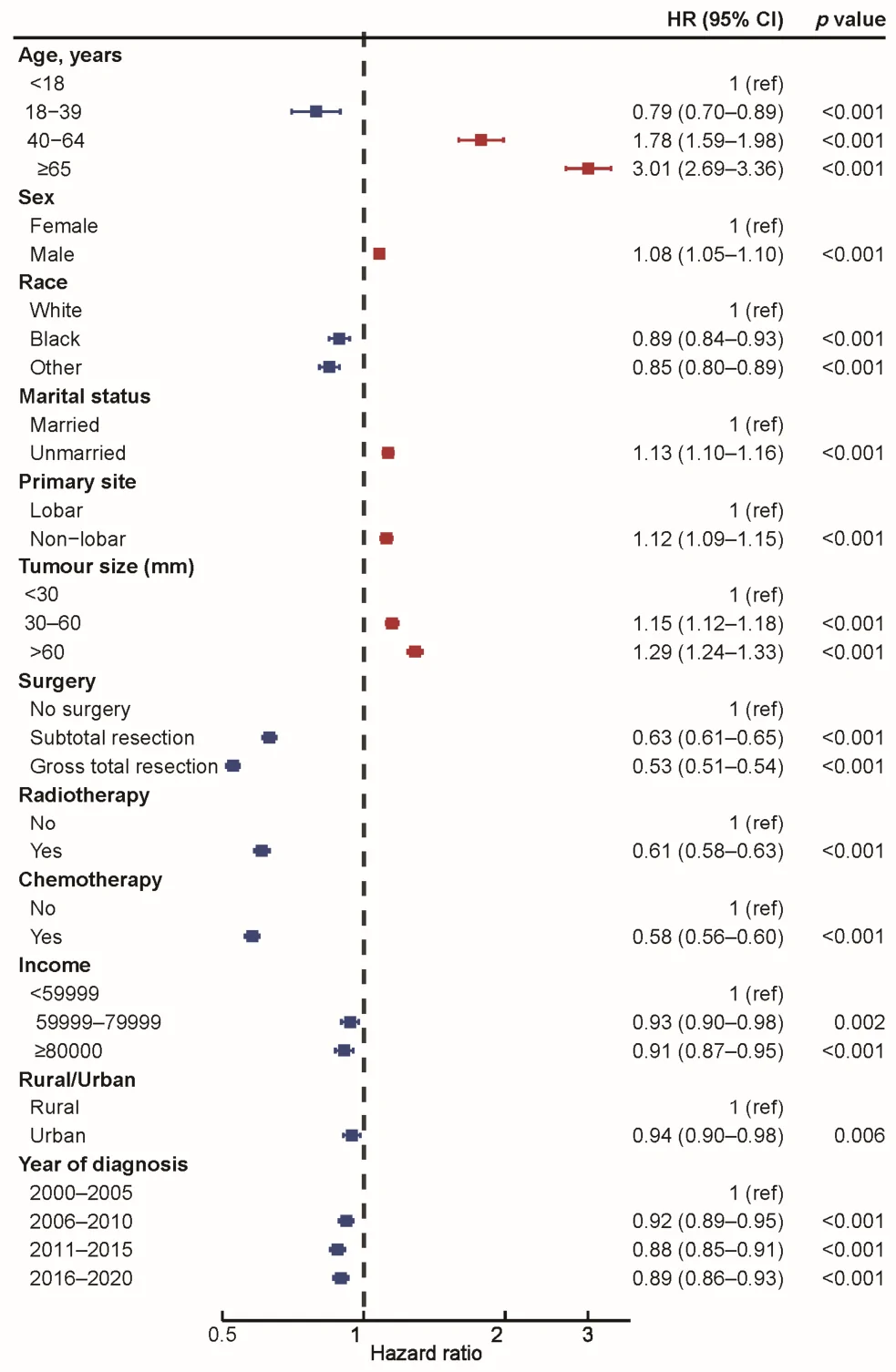
Prognostic factors for overall survival. Forest plot of adjusted hazard ratios (HRs) with 95% CIs for demographic and clinical covariates, including age, sex, race, marital status, primary site, tumor size, surgery, RT, CT, income, rural/urban residence, and year of diagnosis. Reference categories are indicated as “1 (ref)”. HR < 1 favors improved survival.

Multivariable Cox proportional hazards models confirmed that GTR, RT, and CT were independently associated with lower mortality: GTR (HR 0.53, 95% CI 0.51–0.54), RT (HR 0.61, 0.58–0.63), and CT (HR 0.58, 0.56–0.60) (Figure 5). Adverse prognostic factors included male sex (HR 1.08, 1.05–1.10), non-lobar tumor site (HR 1.12, 1.09–1.15), larger tumor size, and unmarried status. Compared to the 2000–2005 period, later diagnostic periods were associated with significantly lower mortality, with hazard ratios ranging from 0.92 (2006–2010) to 0.88 (2011–2015).

## 4. Discussion

In this nationwide cohort study, we found that population-level survival in the USA has improved only modestly and has plateaued in the past decade, despite increasing use of surgery, radiotherapy, and chemotherapy. Median overall survival in our unselected cohort increased from 6.0 months in 2000 to a peak of 10.0 months between 2013 and 2019, with 12-month survival rising from 25% to 43% and 24-month survival from 9% to 19%. These findings are consistent with prior analyses. Koshy and colleagues reported that median relative survival improved from 12 to 15 months and 2-year survival from 15% to 26% among patients treated with surgery and postoperative radiotherapy between 2000 and 2006. These findings closely mirrored the EORTC/NCIC trial, in which radiotherapy plus temozolomide achieved a median survival of 14.6 months and a 2-year survival of 26% [3]. Neth et al. extended these observations to 2017 and showed that median, 12-month, and 24-month survival increased between 2000 and 2011 but then stabilized; for patients diagnosed in 2015–2017, median survival was 11 months, with 12-month and 24-month survival of 45.7% and 19.0%, respectively [16]. Together with our data, these results indicate that the gains achieved with chemoradiotherapy have translated only partially to the real-world GBM population. However, our observational design cannot determine whether the subsequent plateau reflects therapeutic limitations, demographic shifts, or other unmeasured factors.

Interpretation of these long-term survival trends should also consider the substantial evolution in GBM classification during the study period. The 2016 and 2021 WHO classifications increasingly incorporated molecular features, and under the current WHO framework, glioblastoma is restricted to IDH-wildtype tumors, whereas tumors previously classified as IDH-mutant glioblastoma are now designated astrocytoma, IDH-mutant, grade 4 [17]. In addition, substantial molecular heterogeneity within GBM influences prognosis and treatment response [18]. Therefore, temporal changes observed in a registry cohort spanning 2000–2020 may partly reflect evolving diagnostic classification and the underlying molecular case mix, in addition to changes in treatment patterns [19].

We also confirm that newer technologies have, so far, had limited impact on the population level. In the EF-14 trial, adding tumor-treating fields to maintenance temozolomide improved median overall survival from 16.0 to 20.9 months [20]. However, a SEER-based analysis of 27,534 patients treated with surgery and postoperative radiotherapy found that median survival increased only from 14 to 15 months between the 2005–2015 and 2016–2020 periods. The 24-month survival rate changed minimally from 24.7% to 25.6%, with an adjusted hazard ratio of 0.941 for diagnosis after TTF approval [21]. Our finding that overall median survival plateaued around 10 months after 2013, despite increasing chemotherapy use, is concordant with these observations. Nevertheless, the causes of this temporal pattern cannot be established from our registry data and should not be directly attributed to the efficacy of any specific therapy.

Against this background, our most distinctive contribution is the detailed mapping of an age–treatment–survival gradient across the full spectrum of initial treatment combinations. We observed that within every adult age stratum, omission of both radiotherapy and chemotherapy was associated with very poor outcomes (median survival typically 1–5 months), single- or dual-modality regimens produced intermediate survival, and triple therapy (resection, radiotherapy, and chemotherapy) consistently yielded the longest survival. In adults aged 18–40 years, GTR-based multimodal therapy was associated with a median survival of 30.0 months, whereas survival declined to 16.0 months in those aged 56–60 years and 8.0 months in patients aged 85 years or older; by contrast, median survival with no active treatment remained 1–2 months across all age groups. These gradients closely echo those reported in other real-world cohorts. In a French national study of 2053 patients, median survival was 1.8 months after biopsy alone, 5.9 months with regimens not including concurrent radiotherapy–temozolomide and 16.4 months with concomitant radiotherapy–temozolomide. Among patients receiving six or more cycles of adjuvant temozolomide, median survival reached 25.5 months [22]. These data, together with our results, support the notion that intensity and completeness of multimodal therapy are among the strongest modifiable determinants of survival in unselected GBM populations.

At the same time, we show that the use of intensive multimodal regimens declines sharply with age and is patterned by social factors, pointing to substantial undertreatment of vulnerable groups. In our cohort, standard-therapy combinations accounted for more than two-thirds of initial treatment among adults aged 18–40 years but less than one-fifth among those aged 80 years or older. Moreover, the proportion of patients receiving no surgery, radiotherapy, or chemotherapy rose from roughly 5–8% in individuals younger than 65 years to more than one-third in those aged 80 years or older and over half in those aged 85 years or older. These patterns mirror prior US and European work. Dressler et al. reported, in an NCDB analysis of 100,672 patients diagnosed between 1998 and 2011, that 11% received no therapy at all, with these patients more likely to be older, female, Black, or Hispanic; the overall median survival was only 7.5 months, and the best outcomes were observed in those receiving concomitant surgery, chemotherapy, and radiotherapy [23]. In SEER–Medicare data for 4137 patients aged 65 years or older, Iwamoto and colleagues found a median survival of 4 months, with only 61% undergoing resection, 65% receiving radiotherapy, and 10% receiving chemotherapy within 3 months of diagnosis; age, unmarried status, and comorbidities were all associated with reduced odds of receiving radiotherapy or chemotherapy [24]. Our multivariable analyses extend these observations by demonstrating, in a contemporary nationwide cohort, that older age, unmarried status, lower income, and non-lobar tumor location each substantially reduce the likelihood of receiving triple therapy, suggesting that both clinical vulnerability and social context systematically limit access to guideline-concordant care. Future studies incorporating formal interactions between calendar year and socioeconomic factors may help determine whether disparities in access to multimodal therapy have widened or narrowed over time.

The negative correlation between clinical frailty and therapeutic benefit is particularly acute in elderly patients, who account for a growing share of the GBM burden. Chen and colleagues reported that the incidence of glioblastoma among elderly individuals reached 13.16 per 100,000 between 2000 and 2017, with a male-to-female incidence ratio of 1.62 and an overall stable time trend [25]. Randomized evidence indicates that selected older adults can benefit from combined-modality treatment. In the trial by Perry et al., median survival was 9.3 months with hypofractionated radiotherapy plus temozolomide versus 7.6 months with radiotherapy alone (HR 0.67). Among patients with MGMT promoter methylation, median survival was 13.5 versus 7.7 months (HR 0.53) [26]. Contemporary reviews emphasize that performance status and geriatric assessment, rather than chronological age alone, should guide whether elderly patients receive standard chemoradiotherapy, hypofractionated radiotherapy plus temozolomide, temozolomide monotherapy, or radiotherapy alone [27]. In this context, our finding that many older patients receive no tumor-directed treatment and that triple therapy remains uncommon even in relatively younger elderly groups suggests that a substantial proportion of patients who might benefit from evidence-based regimens are not being offered them, although we cannot fully distinguish ineligibility from undertreatment in registry data.

Our results also align with prior work on other prognostic factors and on the limits of current systemic therapy. We found that gross total resection, lobar tumor location, and receipt of radiotherapy and chemotherapy were each independently associated with improved survival. Although the overall analysis suggested a decline in GTR relative to subtotal resection, this trend was driven primarily by the earlier study years, with resection patterns remaining relatively stable after approximately 2010. This registry-level pattern should not be interpreted as declining surgical capability and may instead reflect changes in case mix, tumor characteristics, surgical selection, and SEER coding over time. In a Swedish surgical cohort of 222 patients, Fekete and colleagues reported that median survival increased from 0.73 to 1.07 years between 2004–2008 and 2012–2016, and that MGMT promoter hypermethylation, non-central tumor location, complete resection, good performance status, younger age, and absence of comorbidities were all associated with longer survival [28]. A recent review of temozolomide in GBM highlights that, despite its central role in modern chemoradiotherapy and its ability to increase median survival to approximately 15 months in selected patients, outcomes remain poor and are constrained by MGMT-dependent resistance, DNA repair mechanisms, and treatment-related toxicities [29]. In our study, the absence of data on MGMT status, IDH mutations, detailed radiotherapy parameters, temozolomide dosing and duration, use of tumor-treating fields, performance status, and comorbidities are major limitations that likely contribute to the residual survival heterogeneity we observe across treatment combinations, especially in small subgroups.

A major limitation is the absence of *IDH* mutation and *MGMT* promoter methylation data in SEER. These markers profoundly affect prognosis and chemotherapy response. *IDH*-mutant tumors, though rare, carry better outcomes; their inclusion may inflate survival estimates for some treatment groups. More critically, *MGMT* methylation status determines temozolomide benefit—without it, we cannot ascertain whether the survival gains from chemotherapy are confined to methylated tumors or extend to unmethylated ones. This unmeasured molecular heterogeneity confounds our treatment comparisons, particularly across age groups, where marker prevalence differs. Consequently, our findings describe real-world patterns but cannot inform biomarker-guided therapy. Linking registry data to molecular profiling is essential to refine prognostic and predictive estimates. Immortal time bias may also affect comparisons involving radiotherapy, chemotherapy, and multimodal therapy because patients must survive long enough after diagnosis to receive these treatments [30,31]. Because detailed treatment timing was not available for time-dependent modeling, the observed survival differences should be interpreted as associations rather than causal treatment effects.

These limitations are inherent to registry-based analyses and warrant cautious interpretation of our findings. Our study is observational; treatment selection is non-random and influenced by factors we cannot measure, and immortal time and selection biases cannot be fully excluded. Nevertheless, the age–treatment–survival gradients we describe are large, internally consistent, and concordant with multiple independent datasets from the USA and Europe [1,3,4,7,8,16,21,22,23] supporting their robustness.

## 5. Conclusions

Taken together, our data suggest that improving GBM outcomes will depend not only on novel therapies but also on more equitable and systematic delivery of existing multimodal regimens to patients who are likely to benefit. In practice, this means paying particular attention to older adults, socioeconomically disadvantaged groups, and patients whose social circumstances or tumor location currently limit access to standard care.

## Figures and Tables

**Table 1:** Baseline characteristics of patients diagnosed with glioblastoma (2000–2020).

Characteristic	Overall (*n* = 46,186)
**Age, years**	□
<18	577 (1.3%)
18–39	2611 (5.7%)
40–64	23,115 (50.0%)
≥65	19,883 (43.0%)
**Sex**	□
Female	19,380 (42.0%)
Male	26,806 (58.0%)
**Race**	□
White	40,890 (88.5%)
Black	2610 (5.7%)
Other	2686 (5.8%)
**Marital status**	□
Married	28,633 (62.0%)
Unmarried	15,967 (34.6%)
Unknown	1586 (3.4%)
**Primary site**	□
Lobar	33,166 (71.8%)
Non-lobar	9757 (21.1%)
Unknown	3263 (7.1%)
**Tumor size (mm)**	□
<30	8140 (17.6%)
30–60	21,136 (45.8%)
>60	8105 (17.5%)
Unknown	8805 (19.1%)
**Surgery**	□
No surgery	11,321 (24.5%)
Subtotal resection	14,794 (32.0%)
Gross total resection	20,071 (43.5%)
**Radiotherapy**	□
No	13,019 (28.2%)
Yes	33,167 (71.8%)
**Chemotherapy**	□
No	17,872 (38.7%)
Yes	28,314 (61.3%)
**Household income**	□
<59,999	5690 (12.3%)
59,999–79,999	16,162 (35.0%)
≥80,000	24,331 (52.7%)
Unknown	3 (0.0%)
**Residence**	□
Rural	5638 (12.2%)
Urban	40,548 (87.8%)
**Year of diagnosis**	□
2000–2005	11,293 (24.5%)
2006–2010	10,407 (22.5%)
2011–2015	11,698 (25.3%)
2016–2020	12,788 (27.7%)

**Table 2:** Temporal trends in treatment patterns (2000–2020).

Outcome	OR Per Year (95% CI)	*p*-Value
**Any surgery**	1.03 (1.028–1.036)	<0.001
**Radiotherapy use**	1.01 (1.010–1.017)	<0.001
**Chemotherapy use**	1.08 (1.081–1.088)	<0.001
**Gross total vs. subtotal**	0.92 (0.915–0.922)	<0.001
**GTR + RT + CT**	1.01 (1.005–1.012)	<0.001

**Table 3:** Factors associated with receiving GTR-based multimodal therapy (GTR + RT + CT).

Category	Adjusted OR (95% CI)	*p*-Value
**Age, years**	**□**	**□**
<18	1.00 (ref)	**□**
18–39	1.00 (0.79–1.26)	0.981
40–64	0.73 (0.59–0.91)	0.005
≥65	0.38 (0.30–0.47)	<0.001
**Sex**	**□**	**□**
Female	1.00 (ref)	**□**
Male	1.01 (0.96–1.06)	0.641
**Race**	**□**	**□**
White	1.00 (ref)	**□**
Black	0.87 (0.78–0.97)	0.013
Other	0.84 (0.75–0.93)	<0.001
**Marital status**	**□**	**□**
Married	1.00 (ref)	**□**
Unmarried	0.73 (0.69–0.76)	<0.001
**Household income**	**□**	**□**
<59,999	1.00 (ref)	**□**
59,999–79,999	1.22 (1.12–1.34)	<0.001
≥80,000	1.34 (1.22–1.47)	<0.001
**Residence**	**□**	**□**
Rural	1.00 (ref)	**□**
Urban	0.93 (0.85–1.02)	0.109
**Primary site**	**□**	**□**
Lobar	1.00 (ref)	**□**
Non-lobar	0.50 (0.47–0.53)	<0.001
**Tumor size**	**□**	**□**
<30	1.00 (ref)	**□**
30–60	1.06 (1.00–1.13)	0.051
>60	0.93 (0.86–1.00)	0.043
**Calendar year**	**□**	**□**
Per 1-year increase	0.99 (0.99–1.00)	0.009

## Data Availability

The data are publicly available through the SEER registry (https://seer.cancer.gov/data/ (accessed on 15 January 2026)).

## Supplementary Materials

The following supporting information can be downloaded at https://www.mdpi.com/article/10.3390/cancers18172780/s1: Table S1. Follow-up duration, censoring status, and number of deaths by diagnosis period and age group. Table S2. Overall survival among patients undergoing gross total resection according to diagnosis period.
