# Supplementary material for: Trends in Treatment and Survival Among Patients with Glioblastoma in the United States from 2000 to 2020
Source: Cancers (Basel). 2026 Aug 27;18(17):2780. doi: 10.3390/cancers18172780 (PMC13565623; doi:10.3390/cancers18172780)
Supplement: Supplementary file 1 [file cancers-18-02780-s001.zip › cancers-4490294-supplementary.pdf]

Supplementary Table S1. Follow-up duration, censoring status, and number of deaths by diagnosis period and age group.

**Diagnosis period**	**Age, years**	**N**	**Deaths / uncensored, n (%)**	**Censored, n (%)**	**Median follow-up, months (95% CI)**
**2000–2005**	<18	131	118 (90.1%)	13 (9.9%)	239
	18–39	749	667 (89.1%)	82 (10.9%)	226 (215–234)
	40–64	5,580	5,493 (98.4%)	87 (1.6%)	226 (217–243)
	≥65	4,833	4,815 (99.6%)	18 (0.4%)	237
**2006–2010**	<18	137	118 (86.1%)	19 (13.9%)	164 (158–186)
	18–39	539	473 (87.8%)	66 (12.2%)	165 (158–177)
	40–64	5,426	5,279 (97.3%)	147 (2.7%)	170 (165–175)
	≥65	4,305	4,273 (99.3%)	32 (0.7%)	164
**2011–2015**	<18	174	151 (86.8%)	23 (13.2%)	106 (101–126)
	18–39	607	484 (79.7%)	123 (20.3%)	103 (99–115)
	40–64	5,994	5,729 (95.6%)	265 (4.4%)	111 (107–117)
	≥65	4,923	4,871 (98.9%)	52 (1.1%)	108 (101–135)
**2016–2020**	<18	135	101 (74.8%)	34 (25.2%)	47 (39–53)
	18–39	716	453 (63.3%)	263 (36.7%)	51 (48–55)
	40–64	6,115	5,428 (88.8%)	687 (11.2%)	50 (49–52)
	≥65	5,822	5,570 (95.7%)	252 (4.3%)	49 (44–55)

Supplementary Table S2. Overall survival among patients undergoing gross total resection according to diagnosis period.

Diagnosis period	N	Median OS, months (95% CI)	Deaths, n (%)	Censored, n (%)
2000–2005	6,174	9.0 (9.0–10.0)	6,039 (97.8%)	135 (2.2%)
2006–2010	4,948	12.0 (11.0–12.0)	4,787 (96.7%)	161 (3.3%)
2011–2015	4,155	14.0 (14.0–15.0)	3,943 (94.9%)	212 (5.1%)
2016–2020	4,794	14.0 (14.0–15.0)	4,175 (87.1%)	619 (12.9%)
